# Grey-matter abnormalities in clinical high-risk participants for psychosis

**DOI:** 10.1016/j.schres.2019.08.034

**Published:** 2020-12

**Authors:** Katia Zikidi, Ruchika Gajwani, Joachim Gross, Andrew I. Gumley, Stephen M. Lawrie, Matthias Schwannauer, Frauke Schultze-Lutter, Alessio Fracasso, Peter J. Uhlhaas

**Affiliations:** a Institute for Neuroscience and Psychology, Univ. of Glasgow, UK; b Institute of Health and Wellbeing, Univ. of Glasgow, UK; c Institute of Biomagnetism and Biosignalanalysis, Westphalian Wilhelms University Muenster, Germany; d Department of Psychiatry, Univ. of Edinburgh, UK; e Department of Clinical Psychology, Univ. Edinburgh, UK; f Department of Psychiatry and Psychotherapy, Medical Faculty, Heinrich-Heine University, Düsseldorf, Germany; g Charité Universitätsmedizin, Department of Child and Adolescent Psychiatry, Berlin, Germany

**Keywords:** Clinical high-risk, Psychosis, Grey matter, Magnetic resonance imaging, Neurocognition

## Abstract

The current study examined the presence of abnormalities in cortical grey-matter (GM) in a sample of clinical high-risk (CHR) participants and examined relationships with psychosocial functioning and neurocognition. CHR-participants (n = 114), participants who did not fulfil CHR-criteria (CHR-negative) (n = 39) as well as a group of healthy controls (HC) (n = 49) were recruited. CHR-status was assessed using the Comprehensive Assessment of At-Risk Mental State (CAARMS) and the Schizophrenia Proneness Interview, Adult Version (SPI-A). The Brief Assessment of Cognition in Schizophrenia Battery (BACS) as well as tests for emotion recognition, working memory and attention were administered. In addition, role and social functioning as well as premorbid adjustment were assessed. No significant differences in GM-thickness and intensity were observed in CHR-participants compared to CHR-negative and HC. Circumscribed abnormalities in GM-intensity were found in the visual and frontal cortex of CHR-participants. Moreover, small-to-moderate correlations were observed between GM-intensity and neuropsychological deficits in the CHR-group. The current data suggest that CHR-participants may not show comprehensive abnormalities in GM. We discuss the implications of these findings for the pathophysiological theories of early stage-psychosis as well as methodological issues and the impact of different recruitment strategies.

## 1 Introduction

Abnormalities in grey matter (GM) are an established neuroanatomical feature of schizophrenia (ScZ) that have been disclosed through magnetic resonance imaging (MRI) (Smieskova et al., 2010; Lawrie et al., 1997, Lawrie et al., 1999; Bois et al., 2015; Shenton et al., 2001). Specifically, reductions in GM thickness (Ward et al., 1996; Wright et al., 2000; Kuperberg et al., 2003) have been observed that correlate with certain symptoms of psychosis and neurocognitive impairments (Antonova et al., 2003; Szesko et al., 2003; Zuffante et al., 2001). Meta-analytic studies have indicated that regions most prominently implicated in ScZ are frontal and temporal cortices (Brent et al., 2014; Gur et al., 2007; Honea et al., 2005).

GM-abnormalities in ScZ have been related to excessive pruning of synaptic contacts during adolescence (Feinberg, 1982) as well as aberrant organization of synaptic connections caused by the atypical migration of the neuron precursor cells during gestation (Cannon et al., 2003; Keshavan et al., 1994). Conflicting evidence exists regarding the trajectory of GM-abnormalities. It has been proposed that reductions in GM-volume are progressive during the early stages of ScZ (Shenton et al., 2001; McCarley et al., 1999; Kubicki et al., 2002). However, other studies could not support this finding (Tognin et al., 2014). More recent evidence has also highlighted the possible role of long-term anti-psychotic medication as a contributing factor towards GM-abnormalities in ScZ (Kato et al., 2011; Radua et al., 2012; Fusar-Poli et al., 2013).

An important question is the presence of volumetric and thickness GM-differences in participants at clinical high-risk (CHR) for ScZ. CHR-criteria have been developed based on the presence of attenuated psychotic symptoms (Miller et al., 2003; Yung et al., 2005) as well as in relationship to the basic symptom (BS) concept proposed by Huber and colleagues (Schultze-Lutter et al., 2012). BS involve the presence of self-experienced perceptual and cognitive anomalies that are thought to represent the earliest manifestation of psychosis risk (Schultze-Lutter et al., 2012). Overall, CHR-criteria confer a approximately a 20% risk of developing ScZ within a 2 year period (Fusar-Poli et al., 2016). Moreover, psychosis-risk varies among CHR-subgroups with participants experiencing brief limited intermittent psychotic symptoms (BLIPS) showing the highest transition rates (Kempton et al., 2015;Fusar-Poli et al., 2016).

There is extensive evidence on the presence of GM-deficits in help-seeking CHR-populations with reductions in GM-volume and thickness in PFC, temporal cortex and parietal cortices (Job et al., 2005; Jung et al., 2010; Koutsouleris et al., 2009, Koutsouleris et al., 2010a, Koutsouleris et al., 2010b; Borgwardt et al., 2009). GM-deficits have also been found to correlate with impaired neurocognition (Koutsouleris et al., 2010a, Koutsouleris et al., 2010b) and more pronounced positive symptoms in CHR-cohorts (Iwashiro et al., 2012). Moreover, there is evidence for GM reductions in subcortical areas, such as basal ganglia, vermal and cerebellar lobules (Koutsouleris et al., 2015; Koutsouleris et al., 2012; Wood et al., 2013; De Wit et al., 2017). However, several studies (Klauser et al., 2015; Takahashi et al., 2008; Velakoulis et al., 2006; Ziermans et al., 2009) also failed to find abnormalities in GM in CHR-groups, raising the question whether neuroanatomical deficits are a pervasive feature of emerging psychosis.

Reductions in GM-volume may differentiate between the CHR-participants who convert to psychosis vs. those who do not (Brent et al., 2013; Koutsouleris et al., 2010a, Koutsouleris et al., 2010b; Pantelis et al., 2003). Significant decreases of GM-volume in PFC and temporal cortices as well as in limbic areas and cerebellum were associated with transition to psychosis (Smieskova et al., 2010; Sun et al., 2009; Hunter and Lawrie, 2018). In addition, voxel-based morphometry (VBM) studies further revealed anterior cingulate, parahippocampal and precuneus GM loss among converters (Fusar-Poli et al., 2011). In contrast, there is also evidence for circumscribed GM-increases in CHR-participants who develop psychosis, primarily in limbic areas (Mechelli et al., 2011; Garner et al., 2005).

In the current study, we examined the presence of GM-abnormalities in a sample of 114 CHR-participants that were recruited as part of the Youth Mental Health Risk and Resilience Study (YouR-study) (Uhlhaas et al., 2017). Neuroanatomical abnormalities in psychiatric conditions are often studied using VBM, a structural neuroimaging technique for comparing regional brain volumes differences (Fusar-Poli et al., 2011; Radua et al., 2010; Bora et al., 2012; Honea et al., 2005; Ashburner and Friston, 2000). In VBM analysis, several transformations are applied to the original anatomical data and some of these have been associated with a substantial decrease in the sensitivity to detect GM-abnormalities (Radua et al., 2014). As a result, GM-alterations cannot be easily related to cortical thinning or a decrease in contrast within GM.

In the current study, we employed a whole-brain approach that involved the measurement of GM-intensity in 70 regions of interest (ROIs). We analyzed MRI-data at the individual level with a ROI based approach that preserved participant-specific topology allowing to independently study local GM intensity as well as cortical thickness. Similar approaches have been used in recent studies on in-vivo myelination (Rowley et al., 2017; Hashim et al., 2015).

## 2 Methods

### 2.1 Recruitment and participants

The YouR-Study is a longitudinal study to identify neurobiological and psychological mechanisms and predictors of psychosis-risk (Uhlhaas et al., 2017) and is funded by the Medical Research Council (MRC). CHR-participants were recruited from the community (n = 114) through an online-screening approach (McDonald et al., 2018). In brief, CHR-participants were recruited through a website (see http://www.your-study.org.uk) that identified CHR-participants from the general population through email-invitations, posters and flyers. Specifically, email invitations were sent out to colleges and universities in Glasgow and Edinburgh through which the majority of study participants were identified. Participants then filled out the online-versions of the a) the 16-item Prodromal Questionnaire (PQ-16) (Ising et al., 2012) and b) a 9-item scale of perceptual and cognitive anomalies (PCA) that was developed to assess BS. Participants were invited for clinical interviews if they positively endorsed 6 or more items on the PQ-16 or 3 or more on the PCA. Moreover, n = 8 CHR-participants were recruited from clinical services through referrals.

In addition, a group of participants (n = 39) who did not meet CHR-criteria but were characterized by non-psychotic psychiatric conditions, such as substance abuse and affective disorders, was included.

The positive scale of the Comprehensive Assessment of At-Risk Mental States (CAARMS) (Yung et al., 2005) was administered as well as 14 items of the Schizophrenia Proneness Instrument (SPI-A) (Schultze-Lutter et al., 2007) to determine Cognitive-Perceptive Basic Symptoms (COPER) and Cognitive Disturbances (COGDIS) criteria. CAAMRS/SPI-A interviews were conducted by trained research assistants and MS.c./Ph.D. level-researchers. Moreover, the M.I.N.I. International Neuropsychiatric Interview (M.I.N.I. 6.0) (Sheehan et al., 1998), the scales for premorbid adjustment (Cannon-Spoor et al., 1982) and social and functional role scales (Cornblatt et al., 2007) were administered. Neuropsychological assessments included the Brief Assessment of Cognition in Schizophrenia (BACS), Keefe et al., 2004).

### 2.2 MRI-data collection and pre-processing

#### 2.2.1 Acquisition

MRI-data were acquired from n = 225 participants of which n = 202 participants were used in the final analysis. T1 weighted (T1-w) scans obtained using a 3 T MPRAGE sequence (FoV = 256x256x176 mm3, voxel size =1x1x1 mm3, TR = 2250 ms, TE = 2.6 ms, T1 = 900 ms, FA = 9°).

#### 2.2.2 Processing and segmentation

T1-w volumes were up-sampled to 0.7 mm isotropic to aid the precision of the automatic segmentation. Up-sampling was performed using the AFNI function 3dresample, with linear interpolation (afni.nimh.nih.gov).

Prior to removing the skull, the intensity of T1-w volumes was normalized to remove global inhomogeneities using the AFNI’s 3dUnifize. The skull was removed from each T1-w anatomy using the AFNI’s 3dSkullStrip. The mask obtained was slightly dilated using the AFNI’s 3dmask_tool (dilate_input parameter set to 1) to prevent unwanted erosion of the external portion of GM.

The skull from the un-normalized T1-w volume was removed using the obtained skull stripping mask. At this point the T1-w volume was normalized using AFNI’s 3dUnifize. We performed this normalization step on the skull-stripped data to focus the inhomogeneity correction only within the cerebrum.

**Fig. 1:**
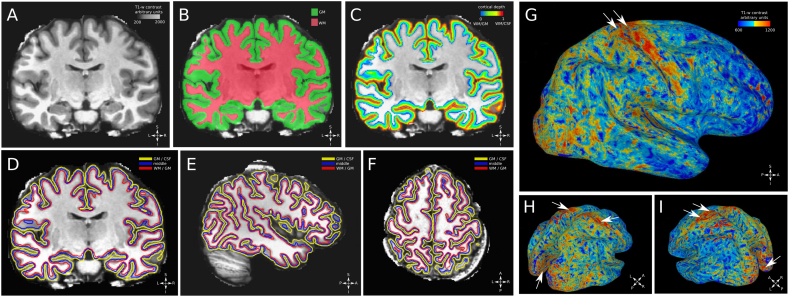
Processing and segmentation. A: T1-w contrast, coronal slice from a single participant, skull removed. B: white matter and grey matter segmentation results, superimposed to a coronal slice, T1-w contrast, single participant. C: equi-volume cortical depth map superimposed to a coronal slice, T1-w contrast. D: white matter/grey matter surface, middle surface and grey matter/cerebro-spinal fluid surface superimposed to a coronal slice, T1-w contrast. E: same as panel D, surfaces superimposed to a sagittal slice, T1-w contrast. F: same as panel D, surfaces superimposed to a horizontal slice, T1-w contrast. G: whole brain surface reconstruction (middle surface), T1-w contrast is mapped over the surface using linear interpolation from a single participant. White arrows indicate motor cortex and somatosensory cortex, showing remarkably high T1-w signal, indicative of the higher myelination of this location compared to neighbouring location (Glasser and Van Essen, 2011, Sereno et al., 2012, Fracasso et al., 2016). H: same as panel G, angled view shows high T1-w signal at the level or the calcarine fissure (white arrows in the posterior part of the brain). White arrows in the superior part of the brain indicate motor cortex and somatosensory cortex. I: same as H, rotated view.

GM and white matter (WM) were segmented on each processed T1-w anatomy using MIPAV v7.4 software (mipav.cit.nih.gov) with the JIST v3.0 (www.nitrc.org/projects/jist/), TOADS-CRUISE vR3c (www.nitrc.org/projects/toads-cruise, Fig. 1A/B).

GM and WM masks were used as input to the volumetric layering tool in CBS-tools (CBS High-Res Brain Processing Tools Version v3.0, www.nitrc.org/projects/cbs-tools). We built an equi-volume map from the WM-GM interface to the GM-cerebrospinal fluid (CSF) interface (Waehnert et al., 2014, see Fig. 1C), providing a normalized reference frame for the location of each voxel across cortical depth from 0 (the WM-GM interface) to 1 (the GM-CSF interface). An estimate of local curvature and cortical thickness was obtained for each voxel within GM.

GM-values of each participant were extracted and detrended for local curvature and cortical thickness (Sereno et al., 2012). The obtained residuals were z-scored and stored in a volume in the same individual T1-w space.

It is important to note that the obtained z-scored GM values were centred on 0, representing the individual T1-w average. Values that positively depart from zero as well as values that negatively depart from zero indicate differences with respect to the mean T1-w, hence higher signal contrast with respect to the mean. On the other hand, values that are closer to zero indicate signal close to the mean T1-w signal, hence lower signal contrast with respect to the mean.

For visualization purposes, 3 surfaces were build based on each T1-w anatomy segmentation, one surface at the WM-GM interface, one surface in the middle of cortical depth and the last surface at the GM-CSF interface (see Fig. 1 D-I). Surfaces were generated from a binarized version of the equi-volume map at the desired level, using the AFNI function IsoSurface.

#### 2.2.3 Atlas co-registration

Each processed T1-w anatomy was co-registered to a standardized talaraich space provided in AFNI (TT_Daemon) using an affine transformation. The probabilistic volumetric Desikan-Killiani atlas (Desikan et al., 2006) was interpolated in each participant T1-w space using nearest-neighbour interpolation. The range of probabilistic values vary between different ROIs, depending on the degree of overlap measured in the original atlas (Desikan et al., 2006). For this reason, we adopted the following criteria to select the voxels in each ROI. For each volumetric ROI and participant, we selected the GM voxels associated with probabilistic values larger than the 25% quantile of the probabilistic ROI itself and located within a cortical depth of 0.4 and 0.6.

From the selected voxels, the average of the detrended z-scored GM values was computed as well as the average local cortical thickness.

#### 2.2.4 Data analysis

*Z*-scored GM and cortical thickness estimates for each participant and ROI were stored in a long-format table, together with the ROI hemisphere (left or right) and demographics variables age and gender and the experimental group (3 levels: controls, CHR and CHR-negative). Furthermore, each ROI was assigned to one of 4 lobes: frontal, parietal, temporal and occipital.

Data analysis on the anatomical dependent variables was performed with a linear mixed effects model implemented in R (www.r-project.org) with the package nlme (Pinheiro et al., 2018). In a linear mixed effect, model the significance of a parameter, or the contrast between two levels in a parameter, is performed using a conditional F-test (Pinheiro and Bates, 2000).

We tested for a modulation of the anatomical dependent variables along the experimental group. The inputs to the model were (as fixed effects): experimental group, lobe, hemisphere, age and gender, allowing for interactions between the terms (hemisphere, age and gender were included as covariates). Each individual participant was modelled as a random effect with a variable intercept.

**Fig. 2:**
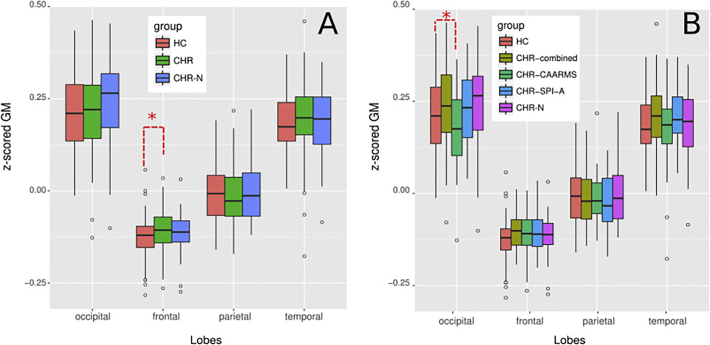
z-scored grey matter (GM) across separate lobes and experimental groups. A: z-scored GM in different lobes for controls (CON), and clinical-high risk (CHR) and CHR-negative (CHR-N) groups. Z-scores are centred on 0, values that depart from zero (either positively or negatively) represent departures with respect to the average T1-w signal. Thus are indicative of higher signal contrast with respect to the average. The occipital lobe shows the highest z-scores as can be expected based on the known high myelination of this location (Glasser and Van Essen, 2011, Sereno et al., 2012, Fracasso et al., 2016); the lowest values can be observed in the frontal lobe. The analysis showed a significant modulation of z-scored GM between the CHR-group and controls in the frontal lobe (see red dashed lines). Other comparisons in different lobes did not survive multiple correction. B: z-scored GM in different lobes for controls (CON), CHR-negative (CHR-N), CHR-CAARMS/SPI-A (CHR_combined), CHR-CAARMS (CHR-CAARMS) and CHR-SPI-A (CHR-SPI-A) groups. The analysis showed a significant modulation of z-scored GM between CHR-CAARMS and controls in the occipital lobe (see red dashed lines). Comparisons in other lobes did not survive multiple correction(see main text for details).

To test for group-differences, we used a separate linear model for each of the 4 lobes (see Fig. 2). The coefficients of the CHR- and CHR-negative were computed relative to the intercept condition of the control group and corrected for multiple comparisons. The same approach was used to examine CHR-subgroups (CAARMS/CHR, SPI-A/CHR, CHR-Combined). Post-hoc analyses were run using *t*-tests (two-tails).

#### 2.2.5 Surface-based visualization

**Fig. 3:**
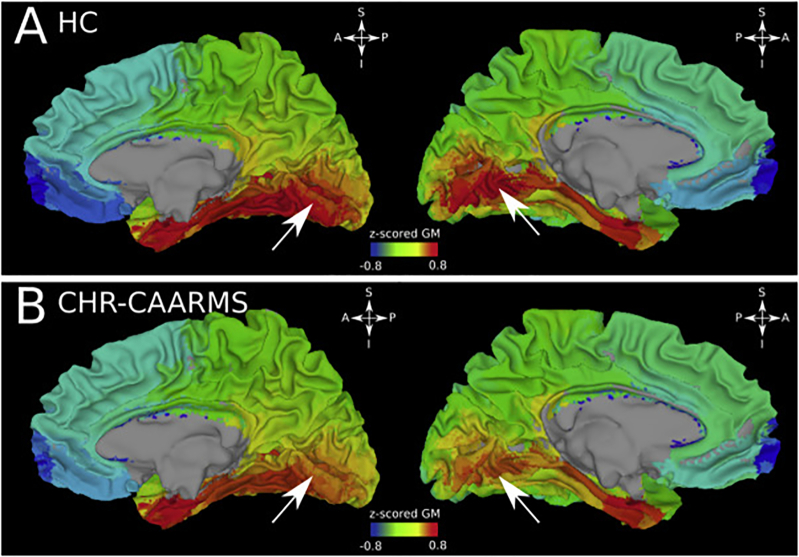
z-scored grey matter (GM) modulation between controls and CHR-CAARMS group in the occipital lobe, standardized surfaces. A: control group, right and left hemispheres, respectively, medial view. High intensity average z-scored are visible at the level of the calcarine fissure (white arrows). B: same as A, but for CHR-CAARMS group. Even though z-scored GM values are higher around the calcarine fissure in this group, overall intensity appears lower compared to the control group (panel A).

The average z-scored GM across participants for each ROI was projected back into the standardized talaraich space. The average z-scored GM was computed for each experimental group. The obtained average z-scored GM volumes were map on a standardized surface using AFNI-SUMA with linear interpolation over surfaces nodes (see Fig. 3).

## 3 Results

### 3.1 Sample characteristics

Of the n = 86 participants which met CAARMS-criteria, n = 84 met APS and 2 genetic/functional decline criteria. The CHR-group had significantly higher CAARMS-positive severity scores, reduced GAF scores as well as lower role and social functioning compared to HC and CHR-negatives (Table 1). There was extensive psychiatric comorbidity in the CHR-group, in particular in regards to affective and anxiety disorders. Compared to the CHR-N group, anxiety, mood disorders, suicide risk, OCD and alcohol dependence/abuse were more pronounced in the CHR-participants.

**Table 1:** Baseline demographic characteristics of CHR, HC and CHR-negative participants.

Characteristic	CHRs (N = 114)	HCs (N = 49)	CHR-Ns (N = 39)	df	F/ X^2^	p	Post-hoc contrast
Age (years), M ± SD	21.68 ± 4.48	22.49 ± 3.57	22.74 ± 4.59	2	F = 1.16	0.31	–
Gender, Female (%)	85 (74.5%)	33 (67.3%)	26 (66.6%)	2	F = 0.68	0.50	–
Years of education, M ± SD	15.27 ± 3.24	16.63 ± 3.03	16.46 ± 3.44	2	F = 3.94	0.02	HCs vs CHRs
GAF, median (range)	58 (21–91)	89 (68–97)	70 (43–84)	2	F = 164.53	<0.001	HCs vs CHR-Ns vs CHRs
CRH-Diagnosis							
CAARMS	35						
SPI-A	28						
CAARMS/SPI-A	51						
CAARMS-Positive Severity, median (range)	28 (0–72)	0 (0−12)	6 (0–24)	2	F = 154.60	<0.001	HCs vs CHR-Ns vs CHRs
GF: Social, median (range)	8 (5–10)	9 (8–9)	8 (6–9)	2	F = 61.35	<0.001	HCs vs CHR-Ns vs CHRs
GF: Role, median (range)	8 (4–9)	9 (5–9)	8 (5–9)	2	F = 27.79	<0.001	HCs vs CHR-Ns vs CHRs
PAS, median (range)							
Childhood	3 (0–16)	1 (0–6)	2 (0–6)	2	F = 24.70	<0.001	HCs vs CHR-Ns vs CHRs
Early adolescence	6 (0−22)	2 (0–8)	4 (0–14)	2	F = 33.44	<0.001	HCs vs CHR-Ns vs CHRs
Late adolescence	5 (0−20)	2 (0−10)	4 (0−13)	2	F = 19.66	<0.001	HCs vs CHR-Ns, HCs vs CHRs
Medication, N (%)	54 (47.3%)		17 (43.5%)	2	X^2^ = 23.31	<0.001	HCs vs CHRs, HCs vs CHR-Ns
None	60 (52.6%)		22 (56.4%)				
Anti-psychotic	1 (0.8%)		0 (0%)	2	F = 0.38	0.68	–
Mood stabiliser	3 (2.6%)		0 (0%)	2	X^2^ = 2.33	0.31	–
Anti-depressant	36 (31.5%)		13 (33.3%)	2	X^2^ = 15.32	<0.001	HCs vs CHRs, HCs vs CHR-Ns
Other	26 (22.8%)		6 (15%)	2	X^2^ = 6.75	0.034	HCs vs CHRs
Diagnosis, N (%)	103 (90.3%)		23 (58.9%)	2	X^2^ = 101.0	<0.001	HCs vs CHR-Ns vs CHRs
Anxiety disorders	94 (82.4%)		17 (43.5%)	2	X^2^ = 92.00	<0.001	HCs vs CHR-Ns vs CHRs
Mood disorders	70 (61.4%)		11 (28.2%)	2	X^2^ = 56.36	<0.001	HCs vs CHR-Ns vs CHRs
Eating disorders	12 (10.5%)		1 (2.5%)	2	X^2^ = 7.36	0.025	HCs vs CHRs
Suicide Risk	57 (50%)		9 (23%)	2	X^2^ = 37.59	<0.001	HCs vs CHRs vs CHR-Ns
Alcohol Dependence/Abuse	32 (28.07%)		10 (25.6%)	2	X^2^ = 11.93	0.003	HCs vs CHRs, HCs vs CHR-Ns
Substance Dependence/Abuse	15 (13.1%)		3 (7.6%)	2	X^2^ = 7.36	0.025	HCs vs CHRs,
Obsessive Compulsive Disorder (OCD)	16 (14.03%)		0 (0%)	2	X^2^ = 13.34	0.001	HCs vs CHRs vs CHR-Ns
Post-Traumatic Stress Disorder (PTSD)	11 (9.6%)		1 (2.5%)	2	X^2^ = 6.66	0.036	HCs vs CHRs
BACS (M/SD)							
Verbal Memory	−0.54/ 1.84	0.12/ 1.37	−0.24/ 1.38	2	F = 2.80	0.06	–
Motor Speed	−0.72/ 1.92	0.07/ 1.21	−0.54/ 1.56	2	F = 3.75	0.02	HCs vs CHRs
Processing Speed	−0.03/ 1.79	0.16/ 1.54	−0.12/ 1.53	2	F = 0.37	0.68	–
Verbal Fluency	0.09/ 1.36	0.41/ 1.37	0.30/ 1.07	2	F = 1.17	0.31	–
Executive Function	−0.07/ 1.89	−0.05/ 1.41	0.12/ 1.02	2	F = 0.21	0.80	–
Working Memory	−0.37/ 1.51	−0.34/ 1.23	−0.37/ 1.98	2	F = 0.007	0.99	–
BACS Composite	−0.46/ 1.58	0.11/ 1.10	−0.21/ 0.99	2	F = 3.13	0.04	HCs vs CHRs

Analyses of neuro-cognitive performance for CHRs, CHR-negatives and HCs revealed significant deficits in motor speed (d = 0.38) as well as for the BACS composite score (d = 0.35) in the CHR-group relative to HC. Moreover, differences between CHR-subgroups were explored (Supplementary Material Table 1).

### 3.2 Neuroanatomical outcomes

GM-intensity: The linear mixed effects model on z-scored GM across CHR-, CHR-negative and HC showed a significant interaction effect between group and lobes (F = 2.25, *p* < 0.05). Specifically, the linear models showed a significant modulation between the CHR-group and HC in the frontal lobe (*T* = 2.73, *p* < 0.01), indicating a decreased T1-w signal contrast in CHR-participants. Effects for occipital, parietal and temporal lobes did not reach statistical significance.

Analysis of CHRs-subgroups revealed an interaction effect between groups and lobes (F = 3.21, *p* < 0.001). The linear model showed a significant modulation of z-scored GM between CHR-CAARMS and HC in the occipital lobe (T = -2.25, p < 0.05), indicating a lower T1-w signal contrast in the CHR-CAARMS group.

To further investigate this difference, we visually inspected the surfaces of visual cortices and performed post-hoc tests. The largest signal modulation was localized around the pericalcarine cortex and the lingual gyrus (T = -2.28, p < 0.05 and T = -2.90, p < 0.01, respectively) and did not extend to neighbouring locations, such as the cuneus, the parahippocalmpal gyrus and the lateral-occipital cortex (T = -0.51, T = -0.83 and T = -1.52, *n.s.*, respectively, see Fig. 3). It is important to note that we used surfaces only for visualization purposes. The statistical analysis is performed on the ROI based analysis on each individual T1-w native space.

Cortical Thickness: There was no significant interaction effect between groups and lobes  for the contrast between CHR-, CHR-negatives and HCs.

Correlations with neuropsychological and demographic variables: We explored systematic correlations between neuropsychological scores, clinical and GM-thickness and intensity by including demographical variables as covariates in a linear regression model. No significant correlations were observed between GM-thickness, GAF, BACS total score and CAARMS-ratings across the whole brain or in any of the four cortical lobes (Supplementary Material Table 2).

Similarly, correlations between GM-intensity, CAARMS-scores and GAF were not significant when corrected for multiple comparisons. However, correlations between GM-intensity and total GAF reached statistical trend levels for the parietal and frontal lobes in CHR-participants (Supplementary Material Table 3).

## 4 Discussion

We examined whole-brain GM-signal intensity and cortical thickness in a CHR-cohort that was largely recruited from the general community. Overall, we did not observe alterations in GM across cortices in CHR-participants. Circumscribed alterations were found in the frontal cortex for the CHR-sample as well as for a subgroup of CHR-participants with attenuated psychotic symptoms in the occipital lobe. In addition, changes in GM-intensity only moderately correlated with neurocognitive impairments.

The decreased T1-contrast in the frontal lobe of CHR-participants could be associated with different processes. Structural abnormalities have been identified in ScZ, particularly in supra-granular layers, involving changes in pyramidal cell density and thinning in the dorso-lateral prefrontal cortex (Jakob and Beckmann, 1994, Kalus et al., 1997, Selemon et al., 1995, Cullen et al., 2006). Moreover, ScZ has been associated with higher neuronal density within GM in frontal and occipital lobes (Selemon et al., 1995).

However, it is not clear how these GM abnormalities observed in ex-vivo samples might transfer to in-vivo T1 contrast. T1 contrast is a complex signal that reflects differences in lipid concentration associated with myelin and is heavily influenced by iron content (Koenig, 1991; Fukunaga et al., 2010; Stueber et al., 2014). Accordingly, it is not possible to identify a single signal source responsible for the observed T1-alteration in the frontal cortex in CHR-participants.

The decreased T1-contrast in the frontal lobe of CHR-participants was not observed when GM-intensity values were examined across CHR-subgroups. Instead, CHR-participants with predominant attenuated psychotic symptoms were characterized by a reduction of GM-intensity values in the occipital cortex, involving the pericalcarine cortex and the lingual gyrus. The lower GM-intensity suggests lower T1-contrast that could potentially index abnormal organization of visual areas.

While phenomenological evidence has highlighted that visual dysfunctions may play a key role in the development of psychosis (Uhlhaas and Mishara, 2006; Klosterkötter et al., 2001), little evidence for anatomical and functional alterations in the visual system exist in CHR-participants (Zhao et al., 2018, Tohid et al., 2015, Nieuwenhuis et al., 2012). This contrasts with a considerable body of work in established ScZ using psychophysical (Yoon et al., 2009; Uhlhaas and Silverstein, 2005; Uhlhaas & Tan et al., 2013), electrophysiological (Javitt et al., 2000; Butler et al., 2001; Grent-T-Jong et al., 2016) as well as anatomical approaches (Dorph-Petersen et al., 2007; Wagstyl et al., 2016) which have highlighted the contribution of aberrant visual processing in the pathophysiology of ScZ.

It should be noted, however, that differences in the visual system were only found in a subgroup of CHR-participants with predominantly attenuated psychotic symptoms, raising the possibility that CHR-participants may be characterized by distinct neurobiological signatures. Future studies need to determine whether there may be stage-specific anatomical alterations during the emergence of psychosis as changes in GM-intensity were not observed in CHR-participants with BS, for example, that also involve alterations in sensory processing that may precede the development of psychotic phenomena (Schultze-Lutter et al., 2012).

The novel observation of circumscribed alterations in GM-intensity in visual cortices may be related to the methodological approach employed in the current study. The majority of studies into GM-changes in CHR-groups and in ScZ have employed a VBM-approach. In VBM analysis, anatomical scans are registered to a standard template and are scaled by the macroscopic transformations to preserve local volumes as well as smoothed to allow for comparison between samples. There is evidence to suggest that this approach may lead to a decrease sensitivity to detect GM-abnormalities (Radua et al., 2014). Moreover, resulting differences can also not be clearly ascribed to cortical thinning or a decrease in contrast within GM.

The approach adopted here differs from standard VBM analysis as T1-data was analyzed at the individual level with an ROI based approach according to the participant-specific topology that could have facilitated the detecting of anatomical changes in visual cortices. Even though the calcarine fissure and the occipito-parietal sulcus can be identified reliably across individuals, their shape are characterized by higher inter-subject variability in cortical folding compared to other brain locations, such as the central sulcus (Tardif et al., 2015). This local variability in cortical folding can negatively influence registration approaches that utilize standardized templates as implemented in VBM, for example, and, as a result affect the sensitivity to detect GM changes in brain location characterized by high inter-subject variability in cortical folding, such as in the visual cortex.

The absence of gross differences in GM in our sample of CHR-participants is consistent with several recent studies that examined GM-values in CHR-populations (Klauser et al., 2015; Ziermans et al., 2010), suggesting that GM-anomalies across cortical regions may not be a pervasive feature of emerging psychosis. Similar to the findings by Klauser et al. (2015), we recruited a sample of CHR-participants who were largely self-referred outside specialised clinics for emerging psychosis. There is evidence that CHR-participants who are not recruited through clinical referral pathways may be characterized by reduced transition rates (Fusar-Poli et al., 2015a, Fusar-Poli et al., 2015b) as well as by subtle differences in the profile of neuropsychological deficits (McDonald et al., 2018). Accordingly, our recruitment approach adopted in this study may have contributed to a CHR-sample characterized by a lower amount of accumulated risk factors for developing psychosis (Fusar-Poli & Schultze-Lutter, 2016; Fusar-Poli et al., 2015a, Fusar-Poli et al., 2015b; Radua et al., 2018). However, follow-up data are needed to address this question.

Our data suggest changes in GM-intensity may only moderately correlate with impairments in neurocognition. Previous studies (Brewer et al., 2006; Lencz et al., 2006; Jung et al., 2010) reported relationships between GM-impairments and impaired cognition in CHR-samples. Interestingly, no significant correlations were observed for correlations with clinical variables, such as psychotic symptom expression.

Finally, the number of female CHR-participants in the current study exceeded previous studies in the field. There is evidence to suggest that anatomical abnormalities may be more pronounced in male CHR-participants (Fusar-Poli et al., 2013) and in male patients with ScZ (Lawrie and Abukmeil, 1998; Suzuki et al., 2002). Accordingly, the large percentage of female CHR-participants may have contributed to the absence of pronounced GM-alterations in the current sample.

## 5 Limitations and Further Steps

We did not assess negative symptoms in CHR-participants which have been shown to impact on GM in CHR-participants (Fusar-Poli et al., 2011). In regard to our analytic approach, several limitations should be acknowledged. The approach we adopted, while participants-specific, relies on the quality of the GM/WM segmentation that provides the basis for the volumetric layering and the cortical thickness computation. Several noise sources as well as inherent anatomical properties can negatively affect the quality of the GM/WM segmentation. These include individual motion in the scanner, poor results of the intensity-based normalization to remove global inhomogeneities and skull-stripping as well as inherent differences in GM contrast between different brain location due to known differences in myelination (Despotovic et al., 2015).

For these reasons, we visually inspect the outcome of each step in the analysis as well as the resulting segmentations. Moreover, we plotted and visually inspected GM-intensity over the cortical surface for each participant to identify well-established patterns due to myelination differences with high T1-w values to facilitate comparisons of visual cortices across participants.

Finally, the current results do not include any follow-up data that could determine whether alterations in GM are predictive for transitioning to psychosis and other outcome parameters in our sample, such as psychosocial functioning. Previous studies (Takahashi et al., 2009; Koutsouleris et al., 2009; Chung and Cannon, 2015, Pantelis et al., 2003) have highlighted that reductions in GM are related to the development of full-blown psychosis as well as continued poor psycho-social functioning in CHR-participants (Koutsouleris et al., 2018).

## 6 Summary

The current study provides further evidence for the absence of gross anatomical differences in CHR-participants. Our novel methodological approach identified, however, circumscribed alterations in frontal and occipital cortices. The latter may highlight the novel contribution of deficits in the visual cortex in sub-groups of CHR-participants that has not been previously described. Follow-up data are required to determine whether changes in GM-intensity and thickness are potential biomarkers for the development of psychosis and continued poor psychosocial functioning in CHR-participants.

## Funding

This study was supported by the project MR/L011689/1 from the 10.13039/501100000265Medical Research Council (MRC).

## Declaration of competing interest

P.J.U. has received research support from Lilly and Lundbeck. S.M.L. has received lecture fees from Janssen, Otsuka and Sunovion. The remaining authors report no biomedical financial interests or potential conflicts of interest.

The following are the supplementary data related to this article.

Supplementary Table 1 Demographic and clinical characteristics of the CHR-Subgroups. Supplementary Table 1

Supplementary Table 2 Cortical thickness correlations. Supplementary Table 2

Supplementary Table 3 GM intensity correlations. Supplementary Table 3

Supplementary data to this article can be found online at https://doi.org/10.1016/j.schres.2019.08.034.
