# Supplementary material for: Grey-matter abnormalities in clinical high-risk participants for psychosis
Source: Schizophr Res. 2020 Dec;226:120–8. doi: 10.1016/j.schres.2019.08.034 (PMC7774586; doi:10.1016/j.schres.2019.08.034)
Supplement: Supplementary Table 1 — Demographic and clinical characteristics of the CHR-Subgroups. [file mmc1.docx]

## Supplementary Table 1: Demographic and clinical characteristics of the CHR-Subgroups.

| Characteristic | CHR-CAARRMS  (N = 35) | | | CHR-SPI-A  (N = 28) | | | CHR-Both  (N = 51) | | | Df | | F/ X^2^ | | | p | Post-hoc contrasts |
| --- | --- | --- | --- | --- | --- | --- | --- | --- | --- | --- | --- | --- | --- | --- | --- | --- |
| Age (years), M ± SD | 20.77± 4.18 | | | 21.46± 3.97 | | | 22.43 ± 4.87 | | | 2 | | F= 1.48 | | | 0.23 | - |
| Gender, Female (%) | 29 (82.8%) | | | 20 (71.4%) | | | 36 (70.5%) | | | 2 | | F= 1.03 | | | 0.36 | - |
| Years of education, M ± SD | 14.63 ± 3.20 | | | 15.11 ± 2.87 | | | 15.80 ± 3.42 | | | 2 | | F= 1.41 | | | 0.24 | - |
| GAF, median (range) | 60 (21-91) | | | 63 (34-91) | | | 52 (21-78) | | | 2 | | F = 7.79 | | | 0.001 | CHR-SPI-A vs CHR-Both |
| CAARMS-Positive Severity, median (range) | 24 (4-63) | | | 11.50 (0-54) | | | 37 (11-72) | | | 2 | | F = 26.87 | | | <0.001 | CHR-CAARMS vs CHR-SPI-A vs CHR-Both |
| GF: Social, median (range) | 8 (5-9) | | | 8 (5-10) | | | 7 (5-9) | | | 2 | | F = 3.56 | | | 0.031 | CHR-SPI-A vs CHR-Both |
| GF: Role, median (range) | 8 (6-9) | | | 8 (6-9) | | | 7 (4-9) | | | 2 | | F = 6.10 | | | 0.003 | CHR-SPI-A vs CHR-Both |
| PAS, median (range) |  |  |  | |  |  | |  |  | |  | |  |  |  |  |
| Childhood | 4 (0-10) | | | 2 (0-16) | | | 3.50 (0-13) | | | 2 | | F = 1.96 | | | 0.14 | - |
| Early adolescence | 6 (1-22) | | | 6 (0-19) | | | 6 (0-19) | | | 2 | | F = 0.15 | | | 0.85 | - |
| Late adolescence | 6 (1-12) | | | 4.50 (0-18) | | | 5 (0-20) | | | 2 | | F = 0.33 | | | 0.71 | - |
| No medication, N (%) | 22 (62.8%) | | | 13 (46.4%) | | | 25 (49.0%) | | |  | |  | | |  |  |
| Medication | 13 (37.1%) | | | 15 (53.5%) | | | 26 (50.9%) | | | 2 | | F = 1.07 | | | 0.34 | - |
| Anti-psychotic | 1 (2.8%) | | | 0 (0%) | | | 0 (0%) | | | 2 | | X^2^ = 2.25 | | | 0.32 | - |
| Mood stabiliser | 1 (2.8%) | | | 0 (0%) | | | 2 (3.9%) | | | 2 | | F = 0.53 | | | 0.58 | - |
| Anti-depressant | 8 (22.8%) | | | 10 (35.7%) | | | 18 (35.2%) | | | 2 | | X^2^ = 1.76 | | | 0.41 | - |
| Other | 5 (14.2%) | | | 7 (25%) | | | 14 (27.4%) | | | 2 | | X^2^ = 2.12 | | | 0.34 | - |
| Diagnosis, N (%) | 33 (94.2%) | | | 24 (85.7%) | | | 46 (90.1%) | | | 2 | | F = 0.64 | | | 0.52 | - |
| Anxiety disorders | 30 (85.7%) | | | 21 (75%) | | | 43 (84.3%) | | | 2 | | F = 0.71 | | | 0.49 | - |
| Mood disorders | 21 (60%) | | | 16 (57.1%) | | | 33 (64.7%) | | | 2 | | F = 0.23 | | | 0.79 | - |
| Eating disorders | 3 (8.5%) | | | 1 (3.5 %) | | | 8 (15.6 %) | | | 2 | | X^2^= 2.99 | | | 0.22 | - |
| Suicide Risk | 21 (60%) | | | 9 (32.1%) | | | 27 (52.9%) | | | 2 | | X^2^= 5.10 | | | 0.07 | - |
| Alcohol Dependence/Abuse | 5 (14.2%) | | | 8 (28.5%) | | | 19 (37.2%) | | | 2 | | X^2^= 5.38 | | | 0.06 | - |
| Substance Dependence/Abuse | 6 (17.1%) | | | 2 (7.1%) | | | 7 (13.7%) | | | 2 | | F = 0.68 | | | 0.50 | - |
| Obsessive Compulsive Disorder (OCD) | 3 (8.5%) | | | 2 (7.1%) | | | 11 (21.5%) | | | 2 | | X^2^= 4.32 | | | 0.11 | - |
| Post-Traumatic Stress Disorder (PTSD) | 1 (2.8%) | | | 1 (3.5%) | | | 9 (17.6%) | | | 2 | | X^2^= 6.72 | | | 0.03 | CHR-CAARMS vs CHR-Both |
