# Supplementary material for: Grey-matter abnormalities in clinical high-risk participants for psychosis
Source: Schizophr Res. 2020 Dec;226:120–8. doi: 10.1016/j.schres.2019.08.034 (PMC7774586; doi:10.1016/j.schres.2019.08.034)
Supplement: Supplementary Table 2 — Cortical thickness correlations. [file mmc2.docx]

**Supplementary Material Table 2. Cortical Thickness Correlations**

|  | GAF Scores | BACS-Composite Score | CAARMS Scores |
| --- | --- | --- | --- |
| **Whole Brain** | .72 | .29 | .62 |
| **Frontal Lobe** | .18 | .34 | .09 |
| **Parietal Lobe** | .40 | .84 | .07 |
| **Temporal Lobe** | .89 | .66 | .84 |
| Occipital Lobe | .80 | .85 | .78 |

*Linear Regression Model outcomes: Uncorrected p-values of correlations between the total BACS Score and Cortical Thickness values across all cortical areas in CHR participants.*
