# Supplementary material for: Grey-matter abnormalities in clinical high-risk participants for psychosis
Source: Schizophr Res. 2020 Dec;226:120–8. doi: 10.1016/j.schres.2019.08.034 (PMC7774586; doi:10.1016/j.schres.2019.08.034)
Supplement: Supplementary Table 3 — GM intensity correlations. [file mmc3.docx]

**Supplementary Material Table 3. GM Intensity Correlations**

|  | GAF Scores | BACS-Composite Score | CAARM Scores |
| --- | --- | --- | --- |
| **Whole Brain** | .89 | .99 | .89 |
| **Frontal Lobe** | .04 | .76 | .66 |
| **Parietal Lobe** | .01 | .15 | .06 |
| **Temporal Lobe** | .35 | .60 | .40 |
| **Occipital Lobe** | .88 | .92 | .38 |
|  |  |  |  |

*Linear Regression Model outcomes: Uncorrected p-values of correlations between the total BACS Score and GM Intensity values across all cortical areas*
